# Structure of a dimeric crenarchaeal Cas6 enzyme with an atypical active site for CRISPR RNA processing

**DOI:** 10.1042/BJ20130269

**Published:** 2013-05-10

**Authors:** Judith Reeks, Richard D. Sokolowski, Shirley Graham, Huanting Liu, James H. Naismith, Malcolm F. White

**Affiliations:** Biomedical Sciences Research Complex, University of St Andrews, North Haugh, St Andrews, Fife KY16 9ST, U.K.

**Keywords:** antiviral defence, Cas6, clustered regularly interspaced short palindromic repeats (CRISPR), ribonuclease, *Sulfolobus*, CRISPR, clustered regularly interspaced short palindromic repeats, Cas, CRISPR-associated, crRNA, CRISPR RNA, Ni-NTA, Ni^2+^-nitrilotriacetate, PaCas6f, *Pseudomonas aeruginosa* Cas6, PfuCas6, *Pyrococcus furiosus* Cas6, RAMP, repeat-associated mysterious protein, RMSD, root mean square deviation, RRM, RNA-recognition motif, SAD, single-wavelength anomalous dispersion, SsoCas6, *Sulfolobus solfataricus* Cas6, TBE, Tris/borate/EDTA, TEV, tobacco etch virus, TtCas6e, *Thermus thermophilus* Cas6

## Abstract

The competition between viruses and hosts is played out in all branches of life. Many prokaryotes have an adaptive immune system termed ‘CRISPR’ (clustered regularly interspaced short palindromic repeats) which is based on the capture of short pieces of viral DNA. The captured DNA is integrated into the genomic DNA of the organism flanked by direct repeats, transcribed and processed to generate crRNA (CRISPR RNA) that is loaded into a variety of effector complexes. These complexes carry out sequence-specific detection and destruction of invading mobile genetic elements. In the present paper, we report the structure and activity of a Cas6 (CRISPR-associated 6) enzyme (Sso1437) from *Sulfolobus solfataricus* responsible for the generation of unit-length crRNA species. The crystal structure reveals an unusual dimeric organization that is important for the enzyme's activity. In addition, the active site lacks the canonical catalytic histidine residue that has been viewed as an essential feature of the Cas6 family. Although several residues contribute towards catalysis, none is absolutely essential. Coupled with the very low catalytic rate constants of the Cas6 family and the plasticity of the active site, this suggests that the crRNA recognition and chaperone-like activities of the Cas6 family should be considered as equal to or even more important than their role as traditional enzymes.

## INTRODUCTION

The CRISPR (clustered regularly interspaced short palindromic repeats) system is an adaptive antiviral defence system found in many bacteria and most archaea. In organisms with an active CRISPR system, invading viral DNA can be captured and incorporated into the genome. This process, known as adaptation, requires the Cas (CRISPR-associated) proteins Cas1 and Cas2 and results in the insertion of a short piece (typically 25–50 bp) of foreign DNA into a region of the genome known as the CRISPR locus [1,2]. The inserted sequence, known as a ‘spacer’, is flanked by direct repeat sequences in an array; a locus can grow to comprise >100 repeat–spacer units. A promoter in the CRISPR locus drives transcription of the array, generating a pre-crRNA (crRNA is CRISPR RNA) that is processed into unit-length mature crRNA species [3–5]. These are loaded into effector complexes, which use the crRNA sequence to detect and degrade invading DNA or RNA during interference, providing immunity from infection (reviewed in [6]).

Three main types of CRISPR system (I, II and III) have been defined on the basis of the presence of the key protein subunits Cas3, Cas9 and Cas10 respectively [7]. These are defined further into specific subtypes, such as type I-A–I-F, depending on the particular proteins present. In type I and III systems, pre-crRNA is processed by the Cas6 endonuclease, whereas type II systems utilize an alternative method involving host RNase III [8]. Cas6 is thus a key component of the majority of CRISPR effector systems. Cas6 proteins typically consist of two tandem RRM (RNA-recognition motif)-like folds {also known as RAMP (repeat-associated mysterious protein) domains [9]} with a diagnostic glycine-rich loop near the C-terminus. Although this core fold is conserved, there are significant differences in Cas6 enzymes from different species. Many, such as TtCas6e (*Thermus thermophilus* Cas6, also known as Cse3) and PaCas6f (*Pseudomonas aeruginosa* Cas6, also known as Csy4) recognize an RNA hairpin structure formed by the CRISPR repeat [10,11]. RNA cleavage typically occurs at the base of the hairpin, whereupon the enzyme remains tightly bound to the product and chaperones it to the effector complex. In most cases, these Cas6 enzymes are integral subunits of the effector complexes and catalyse only single-turnover cleavage of the pre-crRNA [12].

In many of the archaea, CRISPR repeats are not palindromic and are thus unable to form stable hairpin structures [13]. The structure of PfuCas6 (*Pyrococcus furiosus* Cas6) in complex with repeat RNA revealed that the RNA is wound around the outside of the enzyme between the two RAMP domains, analogous to the string around a yo-yo [14]. The pre-crRNA is engaged in a binding cleft on one side of the protein and cleaved in the active site on the other side of the enzyme. However, it is unclear whether this is a general mode for binding unstructured RNA in archaeal Cas6 homologues. For the archaeal type I-A and III-B systems, Cas6 appears to be more loosely associated with the effector complexes [15–17] and should, in theory, be capable of true multiple turnover to generate crRNAs for different clients.

All Cas6 variants studied to date are monomeric proteins with an active-site histidine side chain that is thought to act as a general acid or base [12] during the catalytic cycle. In the present paper, we report the crystal structure and accompanying biochemical data for Cas6 from the crenarchaeon *Sulfolobus solfataricus*. This enzyme has a novel dimeric arrangement that appears to be important for catalysis, as a monomeric variant is significantly less active. Furthermore *S. solfataricus* Cas6, in common with many other crenarchaeal enzymes, lacks the ‘essential’ histidine moiety. This suggests a different mechanism of catalysis, which is probed by site-directed mutagenesis.

## EXPERIMENTAL

### Cloning and site-directed mutagenesis

The *sso1437* gene was amplified by PCR from *S. solfataricus* genomic DNA and cloned into the pET151-TOPO plasmid by TOPO cloning with a cleavable N-terminal His_6_-tag as described previously [18]. Site-directed mutagenesis was performed following standard protocols (QuikChange®, Stratagene). The sequences of oligonucleotides used for cloning and mutagenesis are available from M.F.W. upon request.

### Expression and purification

Sso1437 [SsoCas6 (*S. solfataricus* Cas6)] was expressed in C43(DE3) *Escherichia coli* cells in LB (Luria–Bertani) medium at 37°C until reaching a *D*_600_ of 0.6, followed by induction with 1 mM IPTG (isopropyl β-D-thiogalactopyranoside) and overnight incubation at 25°C. Cell pellets were resuspended in PBS with 1 M NaCl, 10 mM imidazole, 10% glycerol, 9 mM 2-mercaptoethanol, 10 μg·ml^−1^ lysozyme, 0.05 unit·ml^−1^ DNase I and EDTA-free protease inhibitor tablets (Roche). The cells were lysed using a cell disruptor (Constant Systems) at 30000 psi (1 psi=6.9 kPa) and the lysate was cleared by centrifugation at 40000 ***g*** for 45 min at 25°C. The soluble fraction was applied to an Ni-NTA (Ni^2+^-nitrilotriacetate) column (Qiagen), washed with 30 mM imidazole and eluted with 400 mM imidazole. The sample was dialysed into PBS with 1 M NaCl, 10% glycerol and the His_6_-tag cleaved with TEV (tobacco etch virus) protease overnight. The sample was reapplied and washed through the Ni-NTA column in 30 mM imidazole and the cleaved protein was applied to a Superdex™ 75 gel-filtration column (GE Healthcare) equilibrated in 20 mM Tris/HCl (pH 7.5), 1 M NaCl and 10% glycerol. All mutants were purified according to the same protocol.

An (l)-selenomethionine derivative of SsoCas6 was expressed in B834(DE3) *E. coli* cells in M9 medium supplemented with Selenomethionine Nutrient Mix (Molecular Dimensions) and 50 mg·l^−1^ (l)-selenomethionine. The expression protocol was as above except that the cells were harvested after 30 h. The sample was purified as above except that 1 mM 2-mercaptoethanol was added to all buffers.

### Reductive methylation of surface lysine residues

Selenomethionine-labelled SsoCas6 was dialysed into 50 mM Hepes (pH 7.5), 1 M NaCl and 10% glycerol, and the surface lysine residues were reductively methylated using dimethylamine borane complex and formaldehyde as part of the JBS Methylation Kit (Jena Bioscience) according to the manufacturer's instructions. The methylated protein was applied to an S75 column equilibrated in 20 mM Tris/HCl (pH 7.5), 1 M NaCl and 10% glycerol and then concentrated to 8 mg·ml^−1^ for crystallography.

### Structural biology

Optimized crystals of selenomethionine-labelled methylated SsoCas6 were grown under conditions of 0.05 M bicine (pH 9.1) and 28% PEG [poly(ethylene glycol)] 3350 using vapour diffusion with a protein/precipitant ratio of 1:1. The crystal was cryoprotected in mother liquor supplemented with 20% glycerol and flash-cooled in liquid nitrogen. A SAD (single-wavelength anomalous diffraction) dataset was collected on the ESRF (European Synchrotron Radiation Facility, Grenoble, France) ID14-4 beamline at the Se–K absorption edge using a single crystal cooled to 100 K. The data were processed with xia2 [19] using XDS [20] and SCALA [21] and the heavy atom sites located using Phenix AutoSol [22]. The map was improved further using Parrot [23] and the chains were traced using Buccaneer [24], both as part of the CCP4 suite [25]. The structure was refined with cycles of correction in COOT [26] and refinement with REFMAC version 5.6 [27] using TLS (Translation–Libration–Screw-rotation) parameters generated by the TLSMD server [28,29]. The side chains of residues in β_3_ and the β_2_–β_3_ loop were disordered and real-space averaging, using AVE as part of the Uppsala Software Factory [30], was required to assign the residues correctly. Methyl groups were not modelled on to the lysine residues owing to insufficient density. The structure was validated using the MolProbity server [31]. Data collection and refinement statistics are presented in Table 1. The co-ordinates and data for the structure of SsoCas6 were deposited in the PDB under code 3ZFV.

**Table 1:** Data collection and refinement statistics for the structure of selenomethionine-labelled methylated SsoCas6 Data collection statistics are averages with those for the highest resolution shell given in parentheses.

Parameter	SsoCas6
Data processing	
Wavelength (Å)	0.979
Space group	*P*2_1_
*a*, *b*, *c* (Å)	71.7, 127.5, 83.6
α, β, γ (°)	90.0, 110.5, 90.0
Resolution	73.33–2.80 (2.87–2.80)
*R*_merge_	0.07 (0.71)
*I*/σ*I*	23.3 (3.7)
Completeness	99.9 (99.1)
Multiplicity	10.4 (10.2)
Anomalous completeness	99.7 (98.5)
Anomalous multiplicity	5.3 (5.2)
Refinement	
*R*_work_/*R*_free_	0.20/0.24
Mean *B*-value (Å^2^)	
All atoms	33
Protein	33
Water	43
Glycerol	47
RMSD	
Bond lengths (Å)	0.01
Angles (°)	1.45

### RNA oligonucleotide purification, end labelling and marker ladder

The RNA sequence corresponding to the repeat of the C and D CRISPR loci of *S. solfataricus* P2 was ordered from IDT, with the sequence 5′-GAUAAUCUCUUAUAGAAUUGAAAG-3′. The oligonucleotide was gel-purified and end-labelled with [γ-^32^P]ATP as described previously [15]. The RNA marker ladder was generated by alkaline hydrolysis as described previously [16].

### Single-turnover endonuclease assays

SsoCas6 protein (0.5 μM) was incubated with 1–5 nM labelled RNA in nuclease assay buffer [20 mM sodium phosphate (pH 7.5), 100 mM potassium glutamate, 5 mM EDTA and 0.5 mM DTT (dithiothreitol)] at the temperature indicated. At relevant time points, 10 μl samples were removed from the main reaction volume and quenched by addition to 30 μl of pre-distributed acid phenol/chloroform (Ambion), vortex-mixed for ~10 s and centrifuged at 15000 ***g*** for 1 min. Then, 5 μl of the upper aqueous phase was removed and mixed 1:1 with formamide. Samples were heated at 95°C for 2 min immediately before loading on to a pre-warmed 20% denaturing polyacrylamide gel [20% acrylamide, 8 M urea and 1× TBE (45 mM Tris/borate and 1 mM EDTA)] and the products were separated by electrophoresis at 80 W for 90 min in 1× TBE running buffer. Following electrophoresis, gels were scanned by phosphorimaging and analysed using Fuji Imagegauge software as described previously [32].

### Thermofluor assay for protein stability

Protein (SsoCas6 wild-type or SsoCas6-L170D/V202D) (5 μM) was incubated in nuclease assay buffer supplemented with 2× SyproOrange® (Invitrogen). Volumes of 100 μl were accommodated in a 96-well propylene plate (Agilent Technologies) and covered with an optically clear adhesive film (Molecular Dimensions). Plates were spun briefly at 1500 ***g*** before commencing the assay. The temperature was raised from 25 to 95°C in 0.5°C increments, 1 min cycles and the fluorescence levels were monitored in a QPCR System (Stratagene® Mx3005p™) using a FFROX filter set with excitation and emission wavelengths of 492 and 610 nm respectively. Post-assay manipulation of data was undertaken using the DFS analysis (version 2.5) tool developed (and kindly provided) by Niesen et al. [33] (downloadable at ftp://ftp.sgc.ox.ac.uk/pub/biophysics). The inflection point of the sigmoidal monophasic thermal profile of the protein was taken to be the melting temperature (*T*_m_) of the protein [33].

### Calibrated gel filtration

SsoCas6 (the K28H variant, which has the same elution profile as that of wild-type protein) and SsoCas6-L170D/V202D were applied to a HiLoad 26/600 Superdex 200™ gel-filtration column (GE Healthcare) equilibrated in 20 mM Tris/HCl (pH 7.5) and 1 M NaCl. The column was calibrated using gel-filtration standards (Bio-Rad Laboratories) and the molecular masses were estimated as described previously [34].

## RESULTS

### Sso1437: an authentic Cas6 enzyme

**Figure 1:**
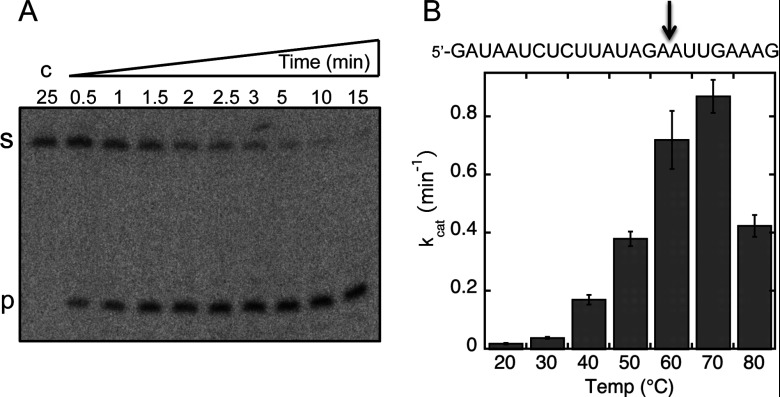
SsoCas6 cleaves a CRISPR RNA repeat (**A**) Representative time course of RNA cleavage by SsoCas6 at 60°C under single-turnover conditions, analysed by gel electrophoresis and phosphorimaging. Labels ‘s’ and ‘p’ indicate substrates and products respectively. The control reaction ‘c’ was carried out in the absence of SsoCas6 for 25 min. (**B**) Single-turnover kinetic rates for cleavage of a CRISPR repeat RNA by SsoCas6 as a function of reaction temperature. The sequence of the RNA oligonucleotide substrate is shown at the top with the cleavage position indicated with an arrow. Each rate was calculated from at least six data points as described in the Experimental section, with means±S.E.M. calculated from curve fitting shown.

The genome of *S. solfataricus* contains five Cas6 paralogues, one of which (Sso2004) was shown previously to be an active ribonuclease by Lintner et al. [15]. Sso2004 shares 90% sequence identity with its paralogue Sso1437, hereafter called SsoCas6. This paralogue was cloned and expressed in *E. coli* with an N-terminal polyhistidine tag. SsoCas6 was purified to homogeneity by a combination of immobilized metal-affinity chromatography and gel filtration, and the polyhistidine tag was removed using TEV protease as described previously [35]. The purified protein was assayed for ribonuclease activity using a ^32^P-end-labelled oligonucleotide corresponding to the CRISPR RNA repeat sequence, 5′-GAUAAUCUCUUAUAGAAUUGAAAG-3′, which matches the CRISPR repeat found in the C and D loci of the *S. solfataricus* P2 genome [36]. Cleavage occurred specifically 8 nucleotides from the 3′ end, generating the conserved ‘5′ handle’ motif [15]. The single-turnover catalytic rate constant for Sso1437 was measured at a range of temperatures from 20 to 80°C (Figure 1). As expected for an enzyme from a thermophile, maximal activity was observed at 70°C, close to the optimum growth temperature of the organism of 80°C. Reaction rates for thermostable enzymes from *S. solfataricus* typically increase 2-fold for each 10°C rise in incubation temperature [37]. This pattern was followed by SsoCas6 up to 60°C, with a modest increase at 70°C and a significant fall in activity thereafter, suggesting that the protein is heat-denatured under these conditions *in vitro* at temperatures above 70°C. The first-order reaction rate of the enzyme *in vivo* at 80°C may thus approach 3–4 min^−1^. SsoCas6 was studied further using a combination of crystallography and site-directed mutagenesis to elucidate its structure and catalytic mechanism.

### The crystal structure of SsoCas6

Although native SsoCas6 crystallized readily, reductive methylation of the surface lysine residues was required to produce well-diffracting crystals. A selenomethionine derivative of the methylated protein was used to solve the structure with a SAD dataset collected to 2.8 Å (1 Å=0.1 nm) resolution. Data collection and refinement statistics are presented in Table 1. Residues 43–50 and 90–93 are disordered in each of the four monomers in the crystallographic asymmetric unit.

**Figure 2:**
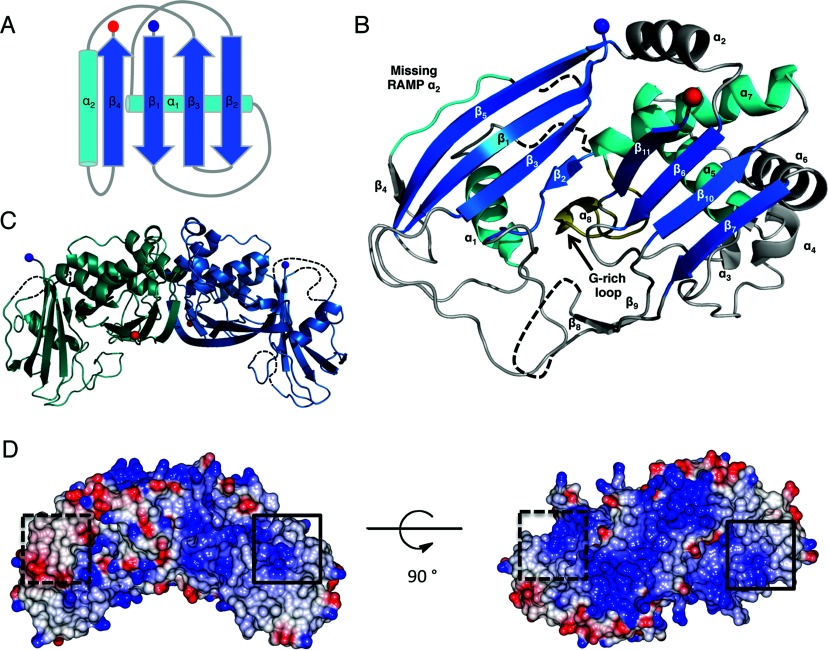
The crystal structure of SsoCas6 (**A**) Schematic representation of a typical ferredoxin-like fold with the β-strands as blue arrows and the α-helices as cyan cylinders. The N- and C-termini are shown as blue and red spheres respectively. (**B**) The structure of SsoCas6 with secondary-structure elements labelled. Disordered loops are shown as broken black lines and the glycine-rich loop shown in yellow. The location of the missing α-helix of the N-terminal domain is indicated. (**C**) View of the SsoCas6 dimer. (**D**) Electrostatic surface potential of the SsoCas6 dimer generated using CCP4MG [50]. The black boxes indicate the active-site region, with the broken box indicating the active site on the non-visible face of the dimer.

Structural homology with other Cas6 proteins was determined with the PDBeFold server [38], which identified the non-catalytic *P. furiosus* Cas6 (PfuCas6nc) as the most structurally similar [PDB code 3UFC, C_α_ RMSD (root mean square deviation) of 3.4 Å over 187 residues], followed by its catalytic paralogue PfuCas6 (PDB code 3I4H, RMSD 3.4 Å over 179 residues). The monomer consists of eleven β-strands and eight helices arranged as two tandem RAMP folds linked by a single helix, consistent with the canonical Cas6 fold [3] (Figures 2A and 2B). RAMP domains are similar to ferredoxin-like and RRM folds, comprising a central four-stranded antiparallel β-sheet (β_4_β_1_β_3_β_2_) flanked on one face by two α-helices (α_1_ and α_2_). The N-terminal RAMP domain of SsoCas6 deviates from the standard motif in that the second RAMP helix (α_2_) that normally precedes β_4_ is missing and replaced by a loop with an extended conformation. In addition to the standard RAMP elements, the N-terminal domain contains a short β-stand located after the missing RAMP α_2_, which forms a small three-stranded antiparallel β-sheet with two strands from the central β-sheet. In the C-terminal RAMP domain, there are three additional helices located on the helical face of the domain as well as a β-hairpin that connects two strands of the central β-sheet. The characteristic glycine-rich loop of the RAMP superfamily is found only in the C-terminal domain and is in the same conformation seen in the other Cas6 homologues.

### Dimerization of SsoCas6

**Figure 3:**
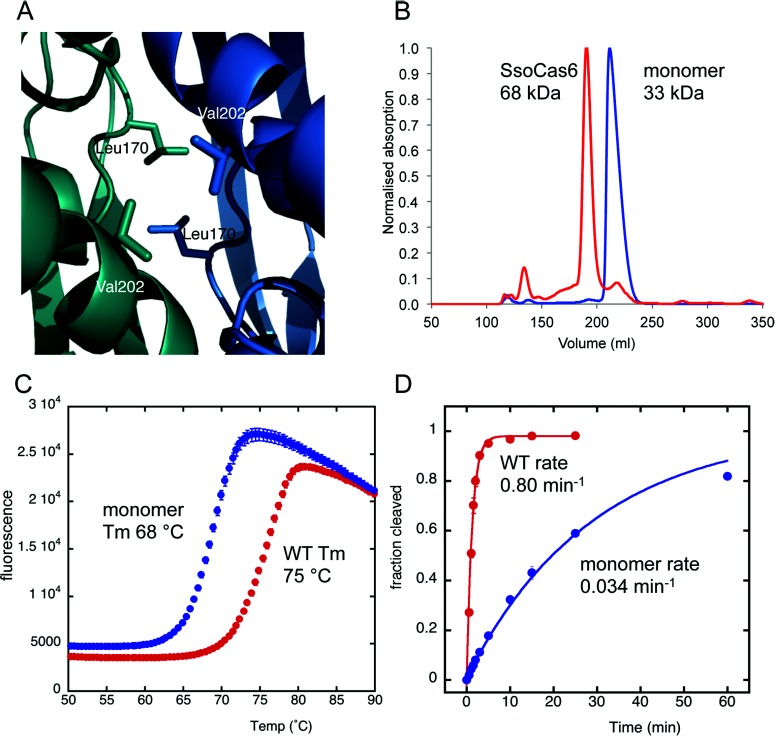
Dimerization of SsoCas6 (**A**) View of Leu^170^ and Val^202^ at the dimer interface. These residues were each changed to aspartate to disrupt the interface. (**B**) Gel-filtration elution profiles of dimeric SsoCas6 and the SsoCas6-L170D/V202D variant, which elutes with a retention volume consistent with a monomeric composition (expected molecular mass of 33 kDa). (**C**) Thermofluor analysis of heat-induced denaturation of wild-type (WT) and monomeric SsoCas6, showing the 7°C difference in melting temperatures (Tm). (**D**) Single-turnover kinetic comparison of wild-type and monomeric SsoCas6. The catalytic activity of the monomer is reduced by 95% compared with the wild-type (WT) enzyme.

Analysis with the PDBePISA server [39] indicates that the four SsoCas6 monomers in the asymmetric unit assemble into two identical dimers (complexation significance score of 1), consistent with gel-filtration data that also suggested a dimer (Figures 2C and 3B). The dimer interface was formed between the C-terminal domains of both proteins with an average buried surface area of 1349 Å^2^. The three non-conserved helices of the C-terminal domain are positioned at the interface and may be responsible for causing dimerization in a Cas6 protein that is typically monomeric. Introduction of charged resides at the centre of the interface (SsoCas6-L170D/V202D, Figure 3A) resulted in a protein that ran on gel filtration as a monomer (Figure 3B). The same dimeric arrangement was seen in low-resolution structures of methylated and non-methylated SsoCas6 crystallized under different conditions (Supplementary Figure S1 at http://www.biochemj.org/bj/452/bj4520223add.htm). This is the first time that a Cas6 protein has been confirmed to be a dimer in the absence of RNA.

The dimeric wild-type enzyme was observed to undergo thermal melting with a melting temperature (*T*_m_) of 75°C. For the monomeric variant, the equivalent *T*_m_ was 68°C. Although 7°C lower than the wild-type enzyme, the monomer remained heat-stable under the assay conditions. The monomeric Cas6 variant had a single-turnover catalytic rate constant of 0.034 min^−1^ at 60°C, equivalent to 4% of the wild-type rate. A similar reduction in rate for the monomeric variant was observed at lower reaction temperatures, suggesting that the loss of activity is not simply due to protein instability (results not shown).

### Delineation of catalytically important residues

The PfuCas6 active site contains a triad of Tyr^31^, His^46^ and Lys^52^ [40]. The Y31A and H46A variants had no detectable enzyme activity, whereas the K52A variant resulted in a 40-fold reduction in nuclease activity. In SsoCas6, the catalytic lysine residue is conserved at position 51 (main chain ordered only in protomer D), whereas Tyr^31^ is not conserved (Supplementary Figure S2 at http://www.biochemj.org/bj/452/bj4520223add.htm). The essential histidine residue, which is also found in more divergent Cas6 orthologues such as Cas6e and Cas6f, is not present in SsoCas6. In fact, there were no histidine residues located around the putative active site. This is also true for the majority of crenarchaeal Cas6 orthologues, suggesting that there may be considerable mechanistic diversity within the archaeal Cas6 family (Supplementary Figure S3 at http://www.biochemj.org/bj/452/bj4520223add.htm).

**Figure 4:**
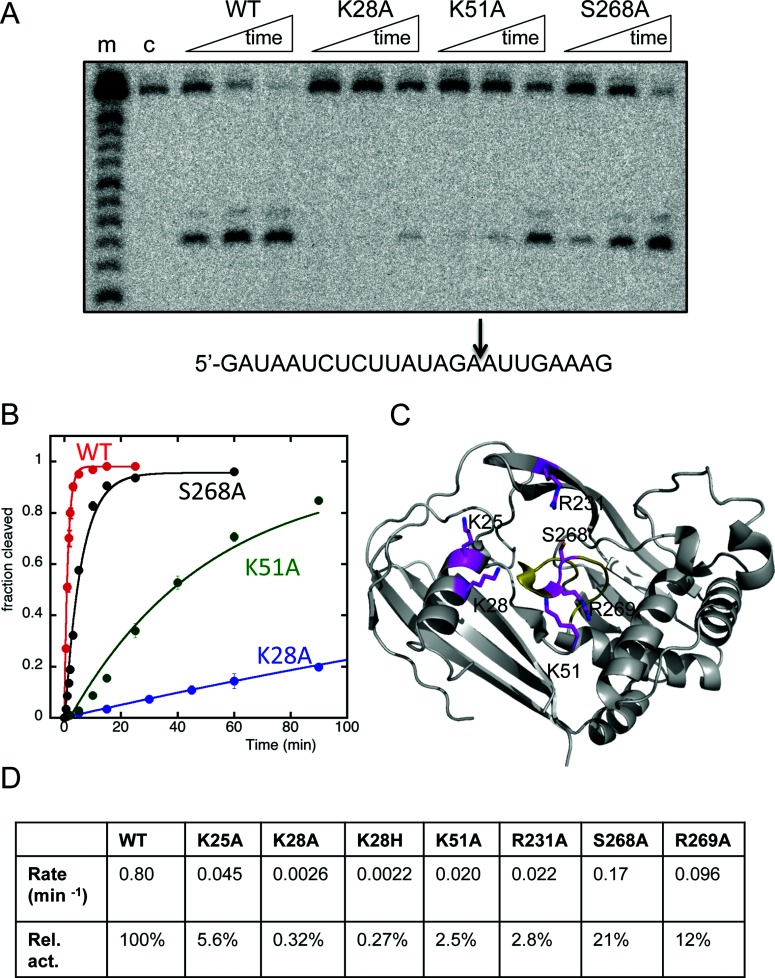
Delineating the SsoCas6 active site (**A**) Phosphorimage of a denaturing polyacrylamide gel showing the reaction products of repeat RNA incubated with wild-type (WT) and selected variant Cas6 enzymes. Lane m shows an RNA ladder generated by alkaline hydrolysis and lane c shows RNA incubated for 60 min in the absence of protein. Time points correspond to 1, 5 and 50 min incubations at 60°C. The cleavage site is indicated in the RNA sequence below the image. (**B**) Plot of the reaction kinetics for selected variants of SsoCas6 (red, wild-type, WT; black, S268A; green, K51A; blue, K28A). All data points were measured in triplicate and are means±S.E.M. (**C**) Structure of the SsoCas6 monomer. The glycine-rich loop is shown in yellow to highlight the approximate position of the active site. The positions of selected side chains targeted by site-directed mutagenesis are shown as magenta sticks. Of these residues, it was not possible to define the absolute conformation of the side chains of Lys^25^, Lys^28^ and Lys^51^ from the electron density. Chains B and D are superimposed on chain A to include all of the desired residues. (**D**) First-order rate constants for wild-type and selected variant SsoCas6 enzymes. Relative activity (Rel. act.) is expressed as a percentage of wild-type (WT) activity.

In order to identify residues important for catalysis, 12 conserved residues present around the putative catalytic site of SsoCas6 were targeted by site-directed mutagenesis (Supplementary Figure S3). The variant proteins were all purified as for the wild-type enzyme and were assayed using a single-turnover kinetic assay with radioactively labelled repeat RNA as a substrate. Under single-turnover conditions at 60°C, wild-type SsoCas6 cleaved the RNA with a catalytic rate constant of 0.80 min^−1^ (Figure 4). Of the variant proteins assayed, the replacement of Lys^28^ with alanine caused the largest reduction in catalytic rate constant (>200-fold), suggesting that this residue, which is strongly conserved in crenarchaeal Cas6 proteins (Supplementary Figure S3), plays an important role in catalysis. The K28H variant had equally low levels of activity, suggesting that a histidine residue cannot substitute for a lysine in this context. Other basic residues around the active site were also investigated. The K25A, K51A, R231A and R269A variants all had catalytic rate constants reduced at least 10-fold compared with the wild-type protein (Figure 4D). The 40-fold decrease in rate observed for K51A compared with the wild-type protein was similar to that observed for the equivalent mutation of K52A in PfuCas6 [40]. Ser^268^, which sits in the highly conserved glycine-rich loop between two conserved arginine residues, was changed to an alanine, but the S268A variant showed only a modest effect on catalytic rate. Changes to other potential active site residues, including T21A, S46A, K161A, Y179F, E192A and S226A, had modest or negligible effects on the activity of the enzyme (results not shown).

## DISCUSSION

### Dimerization of crenarchaeal Cas6 enzymes

The observation that SsoCas6 is a dimer was unexpected. The hydrophobic dimer interface appears to be conserved in the other crenarchaeal Cas6 orthologues, suggesting that this may be a conserved feature of this family (Supplementary Figure S3). In contrast, the other Cas6 enzymes studied to date are all monomeric. This is likely to be a required property for the I-E and I-F systems, where only a single copy of Cas6 is present in the interference complex [41]. A monomeric protein would also ensure the delivery of a single crRNA to the complex. However, for PfuCas6, which is not strongly associated with effector complexes, the paradigm still holds [3]. Dimerization of both the Cas6 of *Pyrococcus horikoshii* and the Cas6b of *Methanococcus maripaludis* has been noted, but only in the presence of pre-crRNA [42,43]. Nevertheless, whereas the monomeric form of SsoCas6 generated in the present study is soluble, the enzyme is much less active than the wild-type dimeric protein. This suggests that the dimeric organization may have some bearing on catalysis, perhaps due to allosteric communication between the two active sites. It is worth bearing in mind that the substrate of Cas6 is a large pre-crRNA with many cleavage sites and that two RNA-cleavage events are required to generate one unit-length crRNA. The dimeric SsoCas6 structure has a broad area of positive charge in the region spanning the dimer interface and the two active sites (Figure 2D). It is possible that pre-crRNA spans this region, allowing both active sites to engage and cleave consecutive recognition sites. This would represent a departure from the simple crRNA wrapping proposed for PfuCas6 [14]. Alternatively, the reduced activity of the monomer may simply be a consequence of its reduced stability or of perturbation of the active site geometry.

### The active site of crenarchaeal Cas6

All Cas6 proteins studied in detail to date have an essential histidine residue in the active site: for example, His^46^ in PfuCas6 [40], His^29^ in PaCas6f [12] and His^26^ in TtCas6e [44]. The exact location of the histidine residue differs in each of the available crystal structures, but is located either in the N-terminal RAMP α_1_ (TtCas6e and PaCas6f) or a non-conserved helix before α_1_ (PfuCas6) (Supplementary Figure S2). In *M. maripaludis* Cas6b, two histidine residues have also been implicated in the catalytic cycle, one of which probably corresponds to His^46^ of PfuCas6 [45]. The role of the catalytic histidine residue was originally thought to be that of a general acid, donating a proton to the leaving group during catalysis [40]. However, in Cas6f, the histidine is deprotonated and acts as a general base, abstracting a proton from the 2′-hydroxy nucleophile during the catalytic cycle [12]. Regardless, the crenarchaeal Cas6 enzymes lack any histidine residues in proximity to the presumed active site, suggesting that these enzymes employ another mechanism. Site-directed mutagenesis suggests that Lys^25^, Lys^28^, Lys^51^ and Arg^231^, which are conserved in crenarchaeal Cas6s (Supplementary Figure S3), are important for catalysis. These residues may play a role in stabilizing the pentacovalent phosphate transition state. Of the four, Lys^28^ appears to be the closest to an essential catalytic residue.

Other residues targeted by mutation had rather modest effects on catalysis. Tyr^179^ is well conserved in the crenarchaeal Cas6 family members and is suitably positioned to participate in catalysis, situated on the right-hand side of the active-site cleft. However, the fact that a phenylalanine residue is well-tolerated at this position suggests that its role may relate to positioning of other side chains or an RNA base. The only conserved acidic residue near the active site, Glu^192^, is clearly not involved in the catalytic cycle as the E192A variant retained full activity. The catalytic site of SsoCas6 thus appears to be unusually resistant to inactivation by targeted mutagenesis, with many highly conserved residues apparently not essential for catalysis. It is possible that many of these residues play a role in crRNA binding, as the single-turnover assay employed in the present study focuses purely on the chemical step of catalysis.

The single-turnover rate constant of SsoCas6, approximately 1 min^−1^ at 60°C, is rather low, although comparable with those of the other Cas6 family enzymes that have been studied [12]. These rates are more akin to those observed for ribozymes than for ribonucleases [46]. However, they are presumably high enough to fulfil the proteins’ functions in pre-crRNA processing *in vivo*, where it is probably more important to be a highly specific ribonuclease than a highly active one. The ease with which crRNA cleavage sites can evolve is exemplified by the type I-C CRISPR system. This subtype lacks a *cas6* gene and instead the Cas5d protein is catalytic, cleaving pre-crRNA *in vitro* [47,48]. A putative catalytic triad consisting of Tyr^46^, Lys^116^ and His^117^ has been identified [47], but these residues are located on a different part of the RAMP domain compared with the active site of Cas6 proteins, suggesting that the active site has evolved independently.

The crystal structure of *S. solfataricus* Cas6 has revealed a dimeric organization that may be relevant for its *in vivo* function as a stand-alone crRNA-processing endonuclease. The paradigm of an ‘active-site histidine residue’ for this enzyme family seems untenable, as no such residue exists within range of the active site. Instead, site-directed mutagenesis has revealed a network of basic residues that each contribute towards catalytic activity without being absolutely essential. The catalytic rate constants of Cas6 family enzymes are so low that they could be considered as much as RNA chaperones as true enzymes. This is particularly relevant for the enzymes that bind hairpin RNA structures very tightly and do not support multiple-turnover catalysis.

While this paper was in revision, Shao and Li [49] reported the crystal structure of a closely related SsoCas6 enzyme in complex with crRNA. This structure is also dimeric and site-directed mutagenesis confirmed the importance of Lys^25^, Lys^28^, Lys^51^ and Arg^232^ for catalysis.

## Online data

Supplementary data
