# Supplementary material for: Structure of a dimeric crenarchaeal Cas6 enzyme with an atypical active site for CRISPR RNA processing
Source: Biochem J. 2013 May 10;452(Pt 2):223–30. doi: 10.1042/BJ20130269 (PMC3652601; doi:10.1042/BJ20130269)
Supplement: Supplementary data [file bj4520223add.pdf]

Biochem. J. (2013) **452**, 223–230 (Printed in Great Britain) doi:10.1042/BJ20130269

SUPPLEMENTARY ONLINE DATA

Structure of a dimeric crenarchaeal Cas6 enzyme with an atypical active site for CRISPR RNA processing

Judith REEKS[1], Richard D. SOKOLOWSKI[1], Shirley GRAHAM, Huanting LIU, James H. NAISMITH[2] and Malcolm F. WHITE[2]
Biomedical Sciences Research Complex, University of St Andrews, North Haugh, St Andrews, Fife KY16 9ST, U.K.

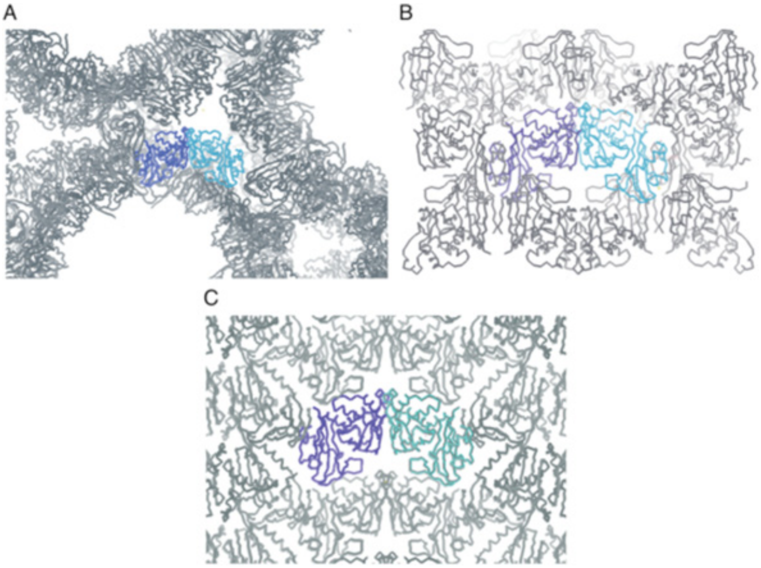

Figure S1 Crystal packing in multiple crystal forms of SsoCas6 showing the conservation of the dimer

Initial molecular replacement (MR) solutions of low-resolution datasets collected from three non-related crystal forms. The data were processed using xia2 [1] and MR was performed using Phaser [2] in the Phenix suite [3] using a monomer of methylated selenomethionine-labelled SsoCas6 as the search model. (**A**) Crystals of SsoCas6 were grown in buffer (20 mM Tris/HCl, pH 7.5, 1 M NaCl and 10 % glycerol) without the use of a precipitant. The crystals were cryoprotected in 75 mM Tris/HCl (pH 7.5), 3 M NaCl and 20 % glycerol. Data were collected on I04-1 (Diamond Light Source) and processed to 4.5 Å resolution with a space group of $F4_132$. The two molecules of the ASU (asymmetric unit) formed the dimer. (**B**) SsoCas6K28A (20 mM Tris/HCl, pH 7.5, 0.5 M NaCl and 10 % glycerol) was incubated with repeat RNA (sequence 5′-GAUAAUCUCUUAUAGAAUUGAAAG-3′, purchased from IDT) at a molar ratio of 1:1.1 and crystallized in 0.1 M Mes (pH 6.8), 0.05 M caesium chloride and 27.5 % Jeffamine M-600. The crystals were cryocooled directly from the drop and diffracted to 3.0 Å resolution on I24 (Diamond Light Source) with a space group of $I222$. No electron density was visible for the RNA. One molecule was present in the ASU, but formed a dimer with a symmetry-related molecule. (**C**) Methylated SsoCas6K28A (20 mM Tris/HCl, pH 7.5, 0.15 M NaCl and 10 % glycerol) was incubated with RNA at a molar ratio of 1:2 and crystallized in 0.1 M Tris/HCl (pH 8.8), 0.2 M $MgCl_2$ and 33 % PEG [poly(ethylene glycol)] 4000 with the drops supplemented with 10 % glycerol. The crystals were cryoprotected with perfluoropolyether. Data were collected on I24 (Diamond Light Source) and processed to 6 Å resolution with a space group of $P4_32_12$. Again, no electron density was observed for the RNA. One molecule was present in the ASU, but formed a dimer with a symmetry mate.

[1] These authors contributed equally to this work.
[2] Correspondence may be addressed to either of these authors (email jhn@st-andrews.ac.uk or mfw2@st-andrews.ac.uk).
The co-ordinates and data for the structure of *Sulfolobus solfataricus* Cas6 have been deposited in the PDB under code 3ZFV.

© The Authors Journal compilation © 2013 Biochemical Society

Biochemical Journal www.biochemj.org

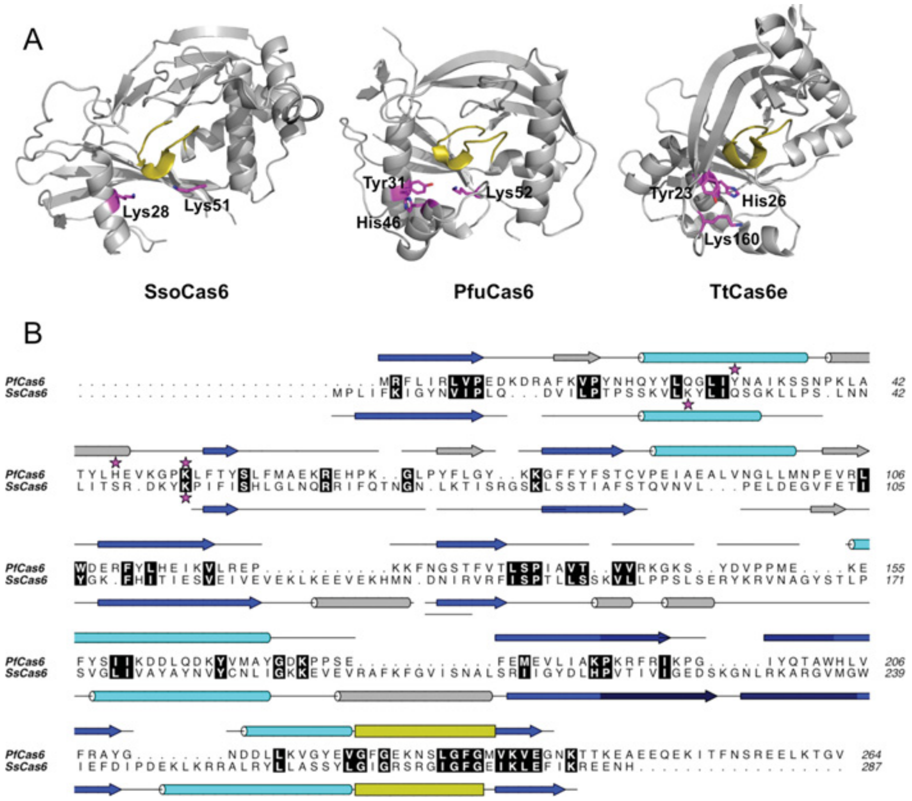

Figure S2 Comparison of Cas6 sequences and structures

(**A**) Structures of (left to right) SsoCas6, PfuCas6 (PDB code 3PKM) and TtCas6e (PDB code 2Y8W) highlighting the positions of the catalytic residues (magenta sticks) and the glycine-rich loop (yellow). (**B**) Structure-based sequence alignment of PfuCas6 and SsoCas6. Sequence similarity is shown in black. Secondary-structure elements are shown above and below the alignment for PfuCas6 and SsoCas6 respectively and are coloured as in Figure S1. Gaps in the connections reflect disordered residues. Putative catalytic residues are indicated with purple stars. The Figure was created with Aline [4].

© The Authors Journal compilation © 2013 Biochemical Society

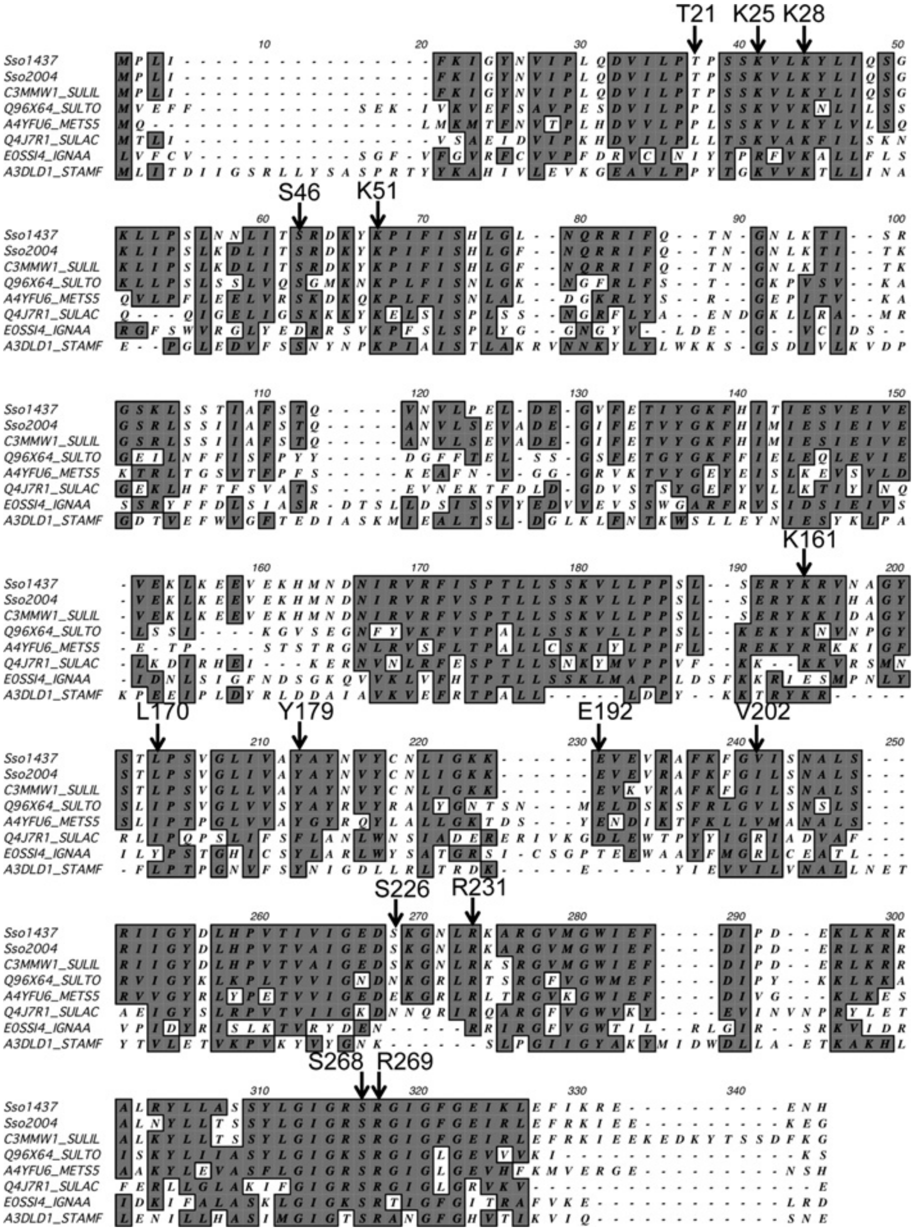

Figure S3 Sequence alignment of crenarchaeal Cas6 proteins

The residues targeted by site-directed mutagenesis are indicated. Representative Cas6 orthologues from *S. solfataricus* (Sso1437 and Sso2004), *Sulfolobus islandicus* (C3MMW1), *Sulfolobus tokodaii* (F9VPJ5), *Metallosphaera sedula* (A4YFU6), *Sulfolobus acidocaldarius* (Q4J7R1), *Ignisphaera aggregans* (E0SS14) and *Staphylothermus marinus* (A3DLD1) are shown. The alignment was generated using T-COFFEE [5].

REFERENCES

1 Winter, G. (2010) xia2: an expert system for macromolecular crystallography data reduction. J. Appl. Crystallogr. **43**, 186–190

2 McCoy, A. J., Grosse-Kunstleve, R. W., Adams, P. D., Winn, M. D., Storoni, L. C. and Read, R. J. (2007) Phaser crystallographic software. J. Appl. Crystallogr. **40**, 658–674

3 Adams, P. D., Afonine, P. V., Bunkoczi, G., Chen, V. B., Davis, I. W., Echols, N., Headd, J. J., Hung, L. W., Kapral, G. J., Grosse-Kunstleve, R. W. et al. (2010) PHENIX: a comprehensive Python-based system for macromolecular structure solution. Acta Crystallogr., Sect. D: Biol. Crystallogr. **66**, 213–221

4 Bond, C. S. and Schuttelkopf, A. W. (2009) ALINE: a WYSIWYG protein-sequence alignment editor for publication-quality alignments. Acta Crystallogr., Sect. D: Biol. Crystallogr. **65**, 510–512

5 Notredame, C., Higgins, D. G. and Heringa, J. (2000) T-Coffee: a novel method for fast and accurate multiple sequence alignment. J. Mol. Biol. **302**, 205–217

Received 22 February 2013; accepted 25 March 2013
Published as BJ Immediate Publication 25 March 2013, doi:10.1042/BJ20130269

© The Authors Journal compilation © 2013 Biochemical Society
